# Monte Carlo-calculated perturbation correction factors in clinical proton beams using PHITS^☆^

**DOI:** 10.1016/j.tipsro.2025.100325

**Published:** 2025-07-25

**Authors:** Hiromu Ooe, Keisuke Yasui, Yuya Nagake, Kaito Iwase, Yuri Kasugai, Mai Tsutsumi, Yuri Fukuta, Shiyu Hori, Hidetoshi Shimizu, Naoki Hayashi

**Affiliations:** a Graduate School of Health Sciences, Fujita Health University, Toyoake, Aichi, Japan; b Division of Medical Physics, School of Medical Sciences, Fujita Health University, Toyoake, Aichi, Japan; c Department of Radiology, Fujita Health University Hospital, Toyoake, Aichi, Japan; d Department of Radiology, Hamamatsu University School of Medicine, Hamamatsu, Shizuoka, Japan

**Keywords:** Proton dosimetry, Beam quality correction factor, Perturbation correction factor, Monte Carlo simulation PHITS

## Abstract

**Background:**

Accurate absolute dosimetry is essential for achieving high-precision proton beam therapy. Consequently, a comprehensive characterization of the ionization chamber’s response properties is necessary.

**Purpose:**

This study aimed to evaluate the average $f_{Q}$ using Monte Carlo (MC) code PHITS to assess uncertainties among different MC simulation tools. Additionally, $P_{Q}$ values for PTW 30013, NACP-02, and PTW 31013 ionization chambers are calculated using PHITS to provide new reference data for $P_{Q}$. Furthermore, a new $k_{Q}$ factor for PTW 31013 chamber is established using MC method, contributing to advancements in proton beam dosimetry protocols.

**Methods:**

Monoenergetic proton beams were employed to calculate $f_{Q}$, $k_{Q}$, and $P_{Q}$ for Farmer, Semiflex, and plane‐parallel chambers. The absorbed dose deposited within the sensitive volume of each chamber was determined via simulations employing PHITS, thereby providing the basis for the estimation of these factors. Computed $f_{Q}$ values were compared with previous reports, while $k_{Q}$ and $P_{Q}$ were benchmarked against literature and Technical Reports Series No. 398 (TRS-398) Rev.1 guideline.

**Results:**

Incorporating PHITS‐derived $f_{Q}$ values reduced the uncertainty of ${\overset{¯}{f}}_{Q}^{PHITS}$ compared to previous findings. The $k_{Q}$ factor for PTW 31013 followed trends observed in cylindrical chambers with varying sensitive volumes; notably, this study represents the first MC estimation of $k_{Q}$ for this chamber. $P_{Q}$ values for values deviated by up to 1.7% from unity.

**Conclusion:**

The data generated in this study provide important insights for refining proton beam dosimetry, contributing to the improvement of treatment precision.

## Highlights

• The number of proton therapy facilities is currently increasing.
• Incorporating $f_{Q}$ values computed using PHITS reduced the uncertainty of ${\overset{¯}{f}}_{Q}^{PHITS}$.
• Variation in $f_{Q}$ and $P_{Q}$ was observed depending on the Monte Carlo code employed.

## Introduction

In absolute dosimetry using an ionization chamber, measurements are performed under conditions that deviate from the ideal assumptions of the Bragg-Gray cavity theory. To address these deviations, a perturbation correction factor ($P_{Q}$) is applied to correct for changes in particle fluence caused by the chamber cavity. $P_{Q}$ is part of the beam quality correction factor ($k_{Q}$), which adjusts for the sensitivity variations of the ionization chamber due to differences between the reference and user beam quality. In the 2024 revision of Technical Reports Series No. 398 (TRS-398 Rev. 1), $P_{Q}$ values for proton beams are provided based on available literature [1]. However, these values are derived from a limited number of Monte Carlo (MC) simulations, such as PENH [2] and Geant4 [3], each employing distinct nuclear interaction models that are challenging to validate experimentally. The uncertainties introduced by discrepancies between these models can be assessed by comparing results from multiple simulation codes.

In previous studies, Gomà and Sterpin [4], Baumann et al. [[5], [6], [7]], Kretschmer et al. [8], and Nagake et al. [9] have calculated $k_{Q}$ and the chamber global factor ($f_{Q}$) for various ionization chambers under monoenergetic proton beams. Additionally, Baumann et al. [10] and Kretschmer et al. [8] determined $P_{Q}$ for different chambers using the MC code Geant4 under similar beam conditions. These investigations indicated that $P_{Q}$ can deviate from unity by up to 1.5%, thereby contributing to the uncertainty in proton dosimetry. Moreover, Baumann et al. [7] used the MC code TOPAS/Geant4 to calculate $k_{Q}$ and $f_{Q}$ for various ionization chambers, finding a maximum deviation of 1.7% compared to results from other MC codes.

Baumann et al. [11] estimated the average $f_{Q}$ ($f_{Q}^{average}$) by calculating the weighted mean of $f_{Q}$ values derived from multiple MC codes, aiming to assess the uncertainty associated with differences in nuclear interaction models among these codes. The simulations were conducted using PENH, Geant4, and FLUKA [12,13]. Their results indicated that the estimated uncertainty was affected by type-B uncertainties, such as discrepancies in physical models, transport parameters, and the geometric modeling of ionization chambers. We propose that incorporating the additional data generated using the Particle and Heavy Ion Transport code and System (PHITS) [14], developed by the Japan Atomic Energy Agency (JAEA), could improve the calculation of the average $f_{Q}$, thereby enabling a more accurate assessment of these uncertainties.

Nagake et al. [9] conducted the Fano test, a method for validating the accuracy of ionization chamber response calculations, using the MC code PHITS, and demonstrated that PHITS offers sufficient precision for such applications. In this study, given both the need to validate results across various nuclear interaction models and the limited use of PHITS in ionization chamber simulations compared to other MC codes, we used PHITS to calculate the parameters $f_{Q}$ and $P_{Q}$ for various ionization chambers, aiming to support high-precision proton beam dosimetry. We recalculated the average $f_{Q}$ by incorporating values from Nagake et al. [9] into the dataset used by Baumann et al. [11], in order to evaluate inter-code uncertainties. To further validate the MC code used in this study, we calculated $P_{Q}$ under monoenergetic proton beam conditions and compared the results with those reported previous studies. Additionally, we extended the dataset for proton beam dosimetry by calculating $f_{Q}$, $P_{Q}$, and MC $k_{Q}$ for PTW 31013 Semiflex ionization chamber, which had not previously been analyzed using MC simulations.

## Methods

Table 1 summarizes the parameter settings and MC simulation configurations used in this study, following the recommendations of American Association of Physicists in Medicine (AAPM) Task Group-268 (TG-268) [15]. Detailed descriptions of these methodologies are provided in the subsequent sections.

**Table 1:** Details of Monte Carlo simulation code used in this study. Nspred and nedisp were set for multi-coulomb scattering and energy straggling modes. Deltc was set for the maximum flight mesh with the energy straggling mode and was set to be divided by the density region.

**Item**	**Description**	
Code version	Particle and Heavy Ion Transport code and System (PHITS) version 3.27	
Hardware	Intel® Core™ i9-9900 K CPU @ 3.60 GHz	
Source Description	Monoenergetic proton source: Between 60 and 250 MeV	
Cross section	nspred (Coulomb scattering of charged particles): 2 (Molière theory)	
	nedisp (Energy straggling during deceleration): 1 (consideration)	
Nuclear reaction model	Initial Stage	: Pearlstein-Nitta
	Dynamic Stage	: Intra-Nuclear Cascade of Liège 4.6
	Static Stage	: generalized evaporation and fission model
Transport parameters	The cut off for all particles was set to 10 keV	
	deltc (The maximum flight mesh with the energy straggling mode): 5 μ m or 10 μ m	
Scored quantities	$D_{air}$: calculated within the chamber’s cavity	
	$D_{w}$: calculated in a region with a radius of 5 mm and a height of 250 μ m	
Statistical uncertainty	Statistical uncertainty was estimated from the standard error of each batch result	
	$D_{air}$ is 0.2% and $D_{w}$ is 0.1%	

### Calculation of $k_{Q}$ and $f_{Q}$ factors

We calculated the beam quality correction factor $k_{Q,\;\;Q_{0}}$ using the following equation [16]:

(1) $$k_{Q,Q_{0}}=\frac{f_{Q}\times W_{air,Q}}{f_{Q_{0}}\times W_{air,Q_{0}}}=\frac{{\left(D_{w}/D_{air}\right)}_{Q}\times W_{air,Q}}{{\left(D_{w}/D_{air}\right)}_{Q_{0}}\times W_{air,Q_{0}}},$$

where Q denotes the user beam quality and $Q_{0}$ represents the reference beam quality used for ionization chamber calibration. When the reference beam quality is ^60^Co γ-rays, the subscript $Q_{0}$ is typically omitted, and the correction factor is denoted simply as $k_{Q}$. $W_{air}$ is the average energy required to produce an ion pair in air at the specified beam quality.

The $f_{Q}$ factor is defined as the ratio of the absorbed dose to water at the reference point ($D_{w}$) to the absorbed dose to air within the ionization chamber cavity ($D_{air}$). In this study, the average $f_{Q}$ derived from PHITS simulations, denoted as ${\overset{¯}{f}}_{Q}^{PHITS}$, was calculated to evaluate the uncertainty among different MC codes under monoenergetic proton beam conditions. The reference beam quality parameter, $f_{Q_{0}}$, was obtained from Andreo et al. [17].

Following the approach by Baumann et al. [11], ${\overset{¯}{f}}_{Q}^{PHITS}$ was calculated as

(2) $${\overset{¯}{f}}_{Q}^{PHITS}\left(E\right)=\frac{f_{Q}^{PENH}\left(E\right)+f_{Q}^{FLUKA}\left(E\right)+\frac{1}{N}\sum_{i=1}^{N}{\left(f_{Q}^{Geant4}\right)}_{i}\left(E\right)+f_{Q}^{PHITS}\left(E\right)}{m},$$

where $f_{Q}^{PENH}\left(E\right)$, $f_{Q}^{FLUKA}\left(E\right)$, $f_{Q}^{Geant4}\left(E\right),$ and $f_{Q}^{PHITS}\left(E\right)$ represent the $f_{Q}$ values obtained from different MC codes in previous studies [4,[6], [7], [8], [9]]. A single value from the literature was used for PENH, FLUKA, and PHITS. In contrast, two literature values were available for Geant4 [7,8] , and these were averaged using a weighting factor of 1/N to account for the unequal number of data points. Here, N denotes the number of $f_{Q}$ factors reported for Geant4, and m is the total number of MC codes used in the calculation.

The uncertainties associated with the calculated ${\overset{¯}{f}}_{Q}^{PHITS}$ were evaluated using the methodology described by Baumann et al. [11]. In accordance with that study, a Gaussian distribution was assumed for the $f_{Q}$ factors.

(3) $$\sigma_{{\overset{¯}{f}}_{Q}^{PHITS}}\left(E\right)=\sqrt{\frac{{\left(f_{Q}^{PENH}\left(E\right)-{\overset{¯}{f}}_{Q}^{PHITS}\left(E\right)\right)}^{2}+{\left(f_{Q}^{FLUKA}\left(E\right)-{\overset{¯}{f}}_{Q}^{PHITS}\left(E\right)\right)}^{2}{+\left(\frac{1}{N}\sum_{i=1}^{N}{\left(f_{Q}^{Geant4}\right)}_{i}\left(E\right)-{\overset{¯}{f}}_{Q}^{PHITS}\left(E\right)\right)}^{2}+{\left(f_{Q}^{PHITS}\left(E\right)-{\overset{¯}{f}}_{Q}^{PHITS}\left(E\right)\right)}^{2}}{m-1}}$$

### Calculation of perturbation correction factors$P_{Q}$

The perturbation correction factor $P_{Q}$ was determined using the following equation:

(4) $$P_{Q}=f_{Q}/{\left(s_{w,air}\right)}_{Q}$$

The same methodology previously applied to photon and electron beams [18,19] was adopted in this calculation. ${\left(s_{w,air}\right)}_{Q}$ denotes the water‐to‐air mass stopping power ratio for beam quality Q. To account for the dependence on beam quality, this ratio was approximated by the following empirical expression:

(5) $${\left(s_{w,air}\right)}_{Q}=a+bR_{res}+\frac{c}{R_{res}},$$

with a=1.131, $b=-2.327\;x\;10^{-5}$, and $c=2.046x10^{-3}$ [1]. The residual range $R_{res}$ was determined via MC calculation.

**Fig. 1:**
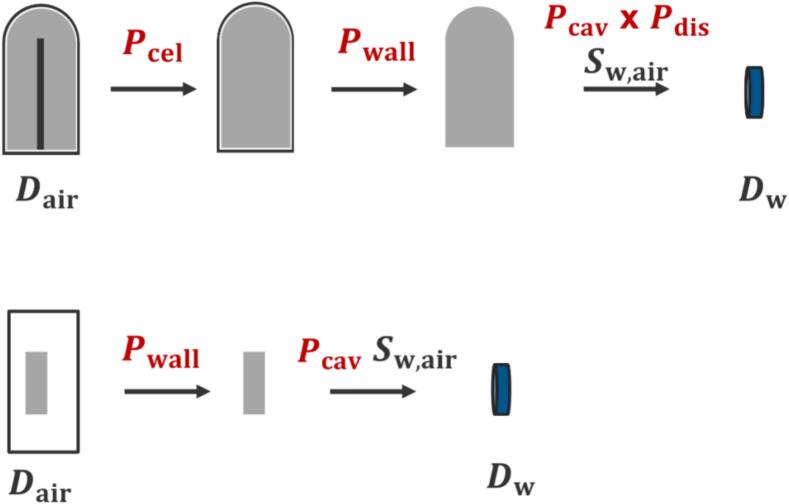
Simulation diagram for determining individual perturbation correction factors for cylindrical and plane-parallel ionization chambers.

Individual perturbation correction factors were determined by sequentially removing specific components of the chamber and calculating the corresponding dose within the chamber cavity. Fig. 1 shows a schematic illustration of this procedure. Based on these individual perturbation correction factors, the overall $P_{Q}$ for cylindrical ionization chambers was calculated as follows:

(6) $$P_{Q}=P_{cel}\times P_{wall}\times P_{stem}\times P_{cav}\times P_{dis}$$

where $P_{stem}$ denotes the correction factor for stem effects, $P_{cav}$ accounts for the perturbation due to the chamber cavity, and $P_{dis}$ represents the displacement correction factor. Consistent with previous studies [10], the stem structure was excluded from the ionization chamber model in this study. This decision was based on the assumption that the contribution of $P_{stem}$ is negligible for cylindrical chambers. The validity of this assumption has been supported by prior sensitivity analyses in the literature, which demonstrated that the stem's influence on the overall perturbation correction factor is minimal. Additionally, this modeling choice enables direct comparison with previous studies that adopted the same approach.

For plane-parallel ionization chambers, $P_{Q}$ has the following equation:

(7) $$P_{Q}=P_{wall}\times P_{cav}$$

### Beam qualities and chamber positioning

To ensure comparability with previous studies [4,[6], [7], [8], [9], [10]], an identical simulation geometry was employed. A 10 × 10 cm^2^ radiation field was oriented perpendicularly to the surface of the water phantom.

Under monoenergetic conditions, we evaluated four energies (150, 160, 200, and 250 MeV) using a Farmer‐type ionization chamber, five energies (70, 100, 150, 200, and 250 MeV) using a Semiflex chamber, and eight energies (60, 70, 80, 100, 150, 160, 200, and 250 MeV) using a plane‐parallel ionization chamber. The ionization chambers were positioned according to the guidelines specified in TRS-398 Rev. 1. For these simulations, the reference depth $z_{ref}$ as 1 g/cm^2^ for low-energy beams (<80 MeV) and 2 g/cm^2^ for high-energy beams (≥80 MeV), in accordance with monoenergetic beam conditions.

### Chamber geometries and materials

**Fig. 2:**
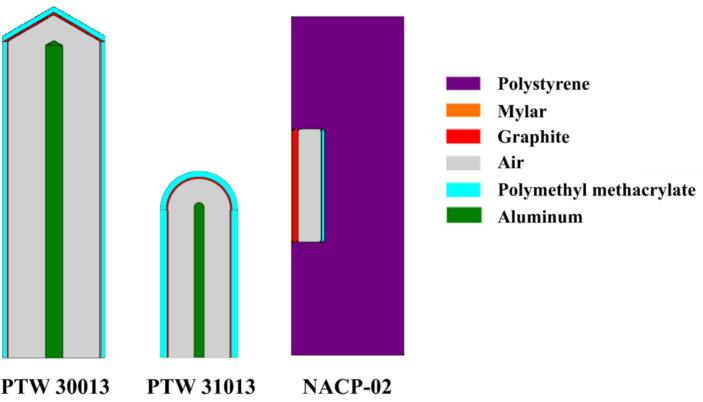
Types of ionization chambers used in this study and their respective construction and materials used. Actual dimensions are not reflected in the figures.

Three types of ionization chambers were analyzed in this study: a Farmer-type chamber (PTW 30013; PTW Freiburg, Freiburg im Breisgau, Baden-Wuerttemberg, Germany), a Semiflex chamber (PTW 31013; PTW Freiburg), and a plane-parallel chamber (NACP-02; IBA Dosimetry, Schwarzenbruck, Bayern, Germany). Detailed structural specifications, materials, and densities for these chambers are provided in Fig. 2 and Table2. Geometric specifications for these ionization chambers were sourced directly from manufacturer-provided technical schematics. The inner wall of PTW 31013 was coated with graphite assigned a density of 0.82 g/cm^3^, as specified in the catalogs from PTW. This low-density graphite likely represents a porous or foam-type material, differing from standard high-density graphite (1.85 g/cm^3^).

**Table 2:** Density (*ρ*) and mean excitation energies (I-value) of materials used. I-value of air, water, and graphite were used from the recommendation of ICRU 90. All the other materials were used from the ATIMA database.

Material	*ρ* (g cm^−3^)	I(eV)
Air	1.20 x 10^-3^	85.7
Water	1.00	78.0
Graphite	1.85	81.0
Graphite Coating	0.82	81.0
Polymethyl Methacrylate (PMMA)	1.19	–
Mylar	1.39	–
Polystyrene	1.05	–
Aluminum	2.70	–

To evaluate uncertainties among different MC codes, the average $f_{Q}$ was re-assessed for the Farmer-type and plane-parallel chambers. In contrast, the Semiflex chamber (PTW 31013) has not previously been examined for $f_{Q}$ or MC $k_{Q}$. To address this gap, comparisons were made among ionization chambers with varying sensitive volumes: PTW 30013 (0.6 cc), PTW 31021 (0.07 cc), and PTW 31022 (0.016 cc), as listed in Table3. The $f_{Q}$ factors for these chambers were derived from prior studies [8,9].

**Table 3:** Details of the ionization chamber structure used for comparison with the $f_{Q}$ of Semiflex ionization chamber under monoenergetic conditions.

	This study	For comparison		
Chambers	PTW 31013	PTW 30013	PTW 31021	PTW 31022
Volume (cc)	0.3	0.6	0.07	0.016
Diameters (mm)	5.5	6.1	4.8	2.9
Length (mm)	15.8	22.9	4.8	2.9

Additionally, the $P_{Q}$ factor of PTW 31013 was evaluated with reference to differences in wall and central electrode materials reported in previous research [8,10]. For other ionization chambers, $P_{Q}$ was compared using the same methodology as that employed by Baumann et al. [10]. Chamber geometry and material composition were based on manufacturer blueprints. Physical densities and mean excitation energies (I-values) used for stopping power calculations were sourced from ICRU90 report [20].

### Monte Carlo code PHITS

The MC code PHITS was employed to calculate the $f_{Q}$ and $P_{Q}$. PHITS has been widely applied in dosimetry and radiation protection studies [[21], [22], [23], [24], [25]]. Nagake et al. [9] confirmed the reliability of PHITS calculations using the Fano test for proton beams, and the parameters were aligned with those in their study. The nuclear reaction process in PHITS was modeled in three stages: the initial stage was described using the Pearlstein–Nitta equation, the dynamic stage employed the Intra-Nuclear Cascade of Liège model [26], and the static stage used a generalized evaporation and fission model. Coulomb scattering of charged particles (excluding electrons and positrons) was treated using Lynch’s equation [27], based on Moliere theory (nspred = 2). Energy straggling during particle deceleration was also considered (nedisp = 1).

## Results and discussion

### $f_{Q}$ factors for monoenergetic proton beams in various ionization chambers

**Fig. 3:**
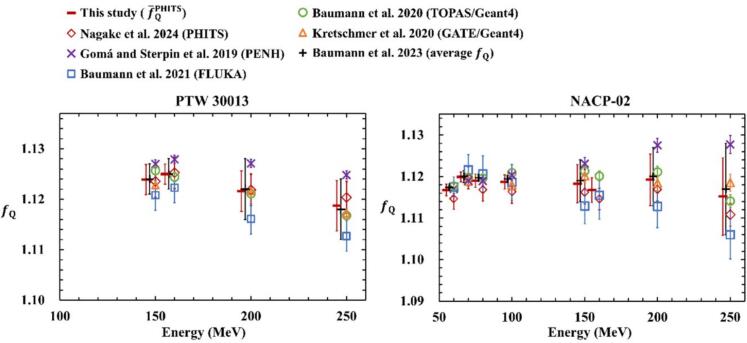
$f_{Q}$ factors calculated using different Monte Carlo codes, the $f_{Q}^{average}$, and ${\overset{¯}{f}}_{Q}^{PHITS}$ in monoenergetic proton beams in the $f_{Q}$ farmer and plane-parallel ionization chambers. The $f_{Q}^{average}$ and ${\overset{¯}{f}}_{Q}^{PHITS}$ are shifted to the left to improve readability.

Fig. 3 shows ${\overset{¯}{f}}_{Q}^{PHITS}$ values obtained in this study. These values were derived by incorporating the $f_{Q}$ factors calculated using PHITS [9], the $f_{Q}^{average}$ reported by Baumann et al. [11], and the $f_{Q}$ factors from previous studies [4,[6], [7], [8], [9]], which were used in computing ${\overset{¯}{f}}_{Q}^{PHITS}$. The error bars represent type-A standard uncertainties. All values referenced from the literature were calculated according to the latest ICRU90 report. Table 4 shows ${\overset{¯}{f}}_{Q}^{PHITS}$ calculated in this study and $f_{Q}$ factors and uncertainties in previous studies. The σ indicates the standard uncertainty.

**Table 4:** ${\overset{¯}{f}}_{Q}^{PHITS}$ calculated in this study and $f_{Q}$ factors and uncertainties reported in previous studies. The σ indicates the standard uncertainty.

$R_{res}$(g cm^−2^)		1.87	2.87	2.99	5.57	13.73	15.64	24.06	36.17
	E(MeV)	60	70	80	100	150	160	200	250
	*z*_ref_ (g cm^-2^)	1	1	2	2	2	2	2	2
Chamber	*s*_w,air_	1.132	1.132	1.132	1.131	1.131	1.131	1.131	1.130
PTW 30013	$f_{Q}^{PENH}$	−	−	−	−	1.1270	1.1279	1.1271	1.1248
	$\sigma_{f_{Q}^{PENH}}$	−	−	−	−	0.0008	0.0008	0.0015	0.0011
	$f_{Q}^{FLUKA}$	−	−	−	−	1.1208	1.1223	1.1161	1.1127
	$\sigma_{f_{Q}^{FLUKA}}$	−	−	−	−	0.0030	0.0031	0.0035	0.0034
	$f_{Q}^{TOPAS/Geant4}$	−	−	−	−	1.1257	1.1244	1.1211	1.1168
	$\sigma_{f_{Q}^{TOPAS/Geant4}}$	−	−	−	−	0.0008	0.0060	0.0060	0.0060
	$f_{Q}^{GATE/Geant4}$	−	−	−	−	1.1228	−	1.1216	1.1172
	$\sigma_{f_{Q}^{GATE/Geant4}}$	−	−	−	−	0.0008	−	0.0008	0.0010
	$f_{Q}^{PHITS}$	−	−	−	−	1.124	1.125	1.122	1.120
	$\sigma_{f_{Q}^{PHITS}}$	−	−	−	−	0.003	0.003	0.003	0.003
	$f_{Q}^{average}$	−	−	−	−	1.124	1.125	1.122	1.118
	$\sigma_{f_{Q}^{average}}$	−	−	−	−	0.003	0.003	0.006	0.006
${\overset{¯}{f}}_{Q}^{PHITS}$**(This study)**		−	−	−	−	**1.124**	**1.125**	**1.122**	**1.119**
$\sigma_{{\overset{¯}{f}}_{Q}^{PHITS}}$**(This study)**		−	−	−	−	**0.003**	**0.002**	**0.004**	**0.005**
NACP-02	$f_{Q}^{PENH}$	1.1170	1.1190	1.1189	1.1203	1.1231	−	1.1275	1.1277
	$\sigma_{f_{Q}^{PENH}}$	0.0011	0.0013	0.0012	0.0014	0.0015	−	0.0017	0.0022
	$f_{Q}^{FLUKA}$	1.1175	1.1216	1.1206	1.1186	1.1129	1.1155	1.1128	1.1060
	$\sigma_{f_{Q}^{FLUKA}}$	0.0024	0.0037	0.0044	0.0043	0.0043	0.0058	0.0051	0.0059
	$f_{Q}^{TOPAS/Geant4}$	1.1177	1.1198	1.1196	1.1209	1.1213	1.1201	1.1211	1.1141
	$\sigma_{f_{Q}^{TOPAS/Geant4}}$	0.0007	0.0008	0.0007	0.0010	0.0012	0.0012	0.0014	0.0015
	$f_{Q}^{GATE/Geant4}$	−	1.1189	−	1.1179	1.1202	−	1.1184	1.1185
	$\sigma_{f_{Q}^{GATE/Geant4}}$	−	0.0009	−	0.0011	0.0016	−	0.0018	0.0021
	$f_{Q}^{PHITS}$	1.115	1.120	1.117	1.116	1.116	1.115	1.117	1.111
	$\sigma_{f_{Q}^{PHITS}}$	0.002	0.002	0.002	0.002	0.,002	0.002	0.002	0.002
	$f_{Q}^{average}$	1.1174	1.1200	1.1197	1.1194	1.119	−	1.120	1.117
	$\sigma_{f_{Q}^{average}}$	0.0004	0.0014	0.0009	0.0009	0.005	−	0.007	0.011
${\overset{¯}{f}}_{Q}^{PHITS}$**(This study)**		**1.1167**	**1.1199**	**1.1190**	**1.1187**	**1.118**	**1.117**	**1.119**	**1.115**
$\sigma_{{\overset{¯}{f}}_{Q}^{PHITS}}$**(This study)**		**0.0014**	**0.0012**	**0.0016**	**0.0017**	**0.005**	**0.003**	**0.006**	**0.009**

For PTW 30013, ${\overset{¯}{f}}_{Q}^{PHITS}$ ranged from 1.119 to 1.125 for proton energies between 150 and 250 MeV, showing a decreasing trend with increasing energy. Baumann et al. [11] reported an $f_{Q}^{average}$ in the range of 1.118–1.125 over the same energy interval, resulting in a discrepancy of less than 0.1%. The maximum standard uncertainty of ${\overset{¯}{f}}_{Q}^{PHITS}$ was 0.5%, which is lower than the uncertainty reported for the $f_{Q}^{average}$ by Baumann et al. [11]. The $f_{Q}$ factors depend on parameters that govern nuclear interactions within each MC code and may vary depending on the type and version of the code used [5,28]. The calculated $f_{Q}$ and $P_{Q}$ values fell within the midrange of previously reported values [4,[6], [7], [8], [9]], indicating that the ${\overset{¯}{f}}_{Q}^{PHITS}$ values contribute to reducing the uncertainty in proton dosimetry.

For NACP-02, ${\overset{¯}{f}}_{Q}^{PHITS}$ showed no energy dependence, which is consistent with the findings of Baumann et al. [11]. The deviations from the $f_{Q}^{average}$ were minimal, with maximum differences of up to 0.2% and a maximum standard uncertainty of 0.8%. At energies below 100 MeV, the $f_{Q}$ factors calculated using PHITS were lower than those obtained with other MC codes, resulting in increased standard uncertainty.

**Fig. 4:**
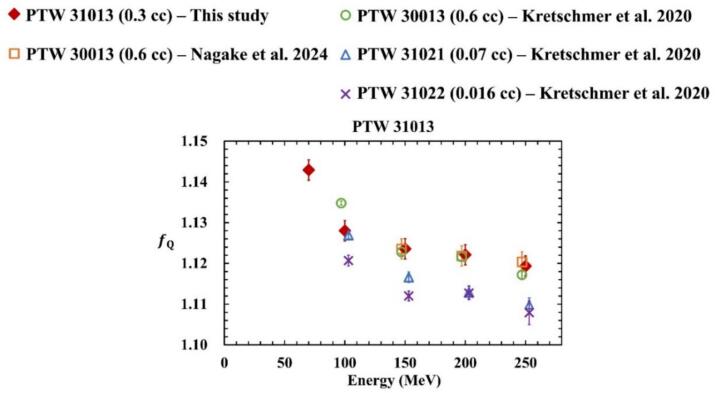
Comparison of $f_{Q}$ using PHITS for PTW 31013 with previous studies using ionization chambers of difference sensitive volume. The $f_{Q}$ factors in previous studies were shifted to the right and left to improve readability.

Fig. 4 shows the $f_{Q}$ factors calculated in this study for the Semiflex ionization chamber (PTW 31013), along with $f_{Q}$ factors reported in the literature [8,9]. For comparison, the $f_{Q}$ factors for the 0.6 cc Farmer (PTW 30013), 0.07 cc Semiflex (PTW 31021), and 0.016 cc PinPoint (PTW 31022) ionization chambers from previous studies [8,9] are also presented. The error bars indicate the standard uncertainty. The values in the literature were calculated according to the latest ICRU90 report.

Over the energy range of 70–250 MeV, the $f_{Q}$ factors ranged from 1.1194 to 1.1429. As energy increased, the $f_{Q}$ factors approached unity, consistent with the findings of previous studies [8,9]. For energies above 150 MeV, the $f_{Q}$ factors differed by less than 0.2% from those reported in earlier investigations using PTW 30013. At 100 MeV, our results agreed within 0.2% with those reported for PTW 31021 [8]. However, above 150 MeV, the discrepancy between the $f_{Q}$ factors for PTW 31021 and PTW 31013 reached 0.9%. This may be due to the larger stem-to-sensitive volume ratio of PTW 31021, which could amplify the stem effect. A similar trend was observed between PTW 31021 and PTW 31022, both of which have smaller sensitive volumes. At 100 MeV, the difference in $f_{Q}$ factors between PTW 31022 and PTW 31013 was 0.7%. Although no cylindrical ionization chamber data were available for 70 MeV, Table 5 shows that the $f_{Q}$ factor for PTW 31013 was higher than that for NACP-02, suggesting that PTW 31013 may not be suitable for measurements below 100 MeV. As illustrated in Fig. 4, deviations in $f_{Q}$ for PTW 31013 become more pronounced at lower energies, especially below 100 MeV. This trend is attributed to increased volume-averaging effects due to steep dose gradients within the chamber cavity, which amplify the impact of $P_{cav}\times P_{dis}$. Therefore, the use of cylindrical ionization chambers like PTW 31013 is not recommended in this energy range, following TRS-398 Rev.1.

**Table 5:** $f_{Q}$ and perturbation correction factor $P_{Q}$ were calculated under monoenergetic conditions. The measured depth and residual range for each energy and the ${\left(s_{w,air}\right)}_{Q}$ used to calculate $P_{Q}$ are shown. The values in parentheses correspond to the standard uncertainty.

$R_{res}$(g cm^−2^)		1.87	2.87	2.99	5.57	13.73	15.64	24.06	36.17
	E(MeV)	60	70	80	100	150	160	200	25
	*z*_ref_ (g cm^-2^)	1	1	2	2	2	2	2	2
Chamber	*s*_w,air_	1.132	1.132	1.132	1.131	1.131	1.131	1.131	1.130
PTW 30013	$P_{cel}$	−	−	−	−	0.999 (±0.003)	0.997 (±0.003)	0.997 (±0.003)	0.999 (±0.003)
	$P_{wall}$	−	−	−	−	0.990 (±0.003)	0.992 (±0.003)	0.989 (±0.003)	0.989 (±0.003)
	$P_{cav}x\;P_{dis}$	−	−	−	−	1.004 (±0.004)	1.007 (±0.004)	1.007 (±0.004)	1.004 (±0.004)
	$P_{Q}$	**−**	**−**	**−**	**−**	**0.994 (±0.004)**	**0.995 (±0.004)**	**0.992 (±0.004)**	**0.991 (±0.004)**
PTW 31013	$f_{Q}$	**−**	**1.143 (±0.003)**	**−**	**1.128 (±0.003)**	**1.124 (±0.003)**	**−**	**1.122 (±0.003)**	**1.119 (±0.003)**
	$P_{cel}$	−	0.996 (±0.003)	−	0.998 (±0.003)	0.996 (±0.003)	−	0.997 (±0.003)	0.998 (±0.003)
	$P_{wall}$	−	0.992 (±0.003)	−	0.994 (±0.003)	0.992 (±0.003)	−	0.995 (±0.003)	0.990 (±0.003)
	$P_{cav}x\;P_{dis}$	−	1.022 (±0.004)	−	1.005 (±0.004)	1.005 (±0.004)	−	1.001 (±0.004)	1.002 (±0.004)
	$P_{Q}$	**−**	**1.010 (±0.004)**	**−**	**0.997 (±0.004)**	**0.994 (±0.004)**	**−**	**0.993 (±0.004)**	**0.990 (±0.004)**
NACP-02	$P_{wall}$	0.983 (±0.003)	0.995 (±0.003)	0.993 (±0.003)	0.992 (±0.003)	0.989 (±0.003)	0.991 (±0.003)	0.993 (±0.003)	0.990 (±0.003)
	$P_{cav}$	0.989 (±0.004)	0.994 (±0.004)	0.994 (±0.004)	0.995 (±0.004)	0.998 (±0.004)	0.994 (±0.004)	0.995 (±0.004)	0.993 (±0.004)
	$P_{Q}$	**0.985 (±0.004)**	**0.989 (±0.004)**	**0.987 (±0.004)**	**0.987 (±0.004)**	**0.987 (±0.004)**	**0.986 (±0.004)**	**0.988 (±0.004)**	**0.983 (±0.004)**

**Fig. 5:**
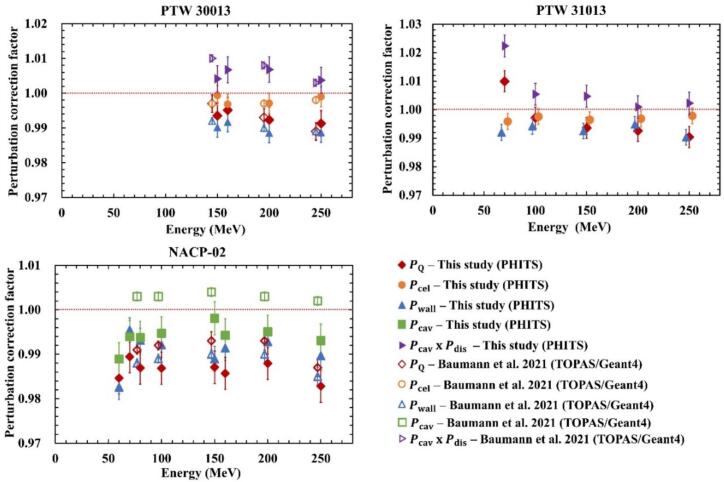
Perturbation correction factors $P_{cel}$, $P_{wall}$, $P_{cav}$, $P_{cav}xP_{dis},$ and $P_{Q}$ for cylindrical and plane-parallel ionization chambers in monoenergetic proton beams.

Fig. 5 shows the $P_{Q}$ factors calculated in this study for various ionization chambers (PTW 30013, PTW 31013, and NACP-02), along with $P_{Q}$ factors reported in the literature [10]. The error bars indicate standard uncertainties. Since this study provides the first detailed parameter calculation for PTW 31013, no literature data for this model are included in the figure.

Fig. 5 (a, b) shows the $P_{cel}$, $P_{wall}$, $P_{cav}xP_{dis}$, and $P_{Q}$ for cylindrical ionization chambers. Deviations of up to 0.4% for $P_{cel}$ and 1.2% for $P_{wall}$ were observed, consistent with the uncertainty margins reported in a previous study using TOPAS/Geant4 [10]. For PTW 31013, a slight energy dependence was observed, with $P_{Q}$ gradually decreasing as energy increased. At 70 and 100 MeV, the product $P_{cav}\times P_{dis}$ was notably affected by volume-averaging due to dose gradients and the chamber’s sensitive volume, leading to an increase in $P_{Q}$. $P_{cel}$ deviated from unity by up to 0.4%, and $P_{wall}$ by up to 1.0%.

Fig. 5 (c) shows $P_{wall}$, $P_{cav}$, and $P_{Q}$ for plane-parallel ionization chamber NACP-02. The trend contrasts with that observed in cylindrical chambers in previous studies [10], where reported no energy dependence. Table 6, Table 7 summarize the characteristics of various cylindrical chambers, including chamber types, wall and central electrode materials, perturbation factors $P_{wall}$ and $P_{cel}$, and maximum deviations from unity. The wall and central electrode materials were chosen based on previous studies [8,10] and manufacturer design drawings. As shown in Table 6, aluminum (density 2.70 g/cm^3^) is most commonly used as the central electrode material. The deviation of $P_{cel}$ from unity increases as the sensitive volume of the ionization chamber decreases, and these deviations appear largely independent of chamber type. Table 7 indicates that polymethyl methacrylate (PMMA, density 1.19 g/cm^3^) and graphite (density 1.85 g/cm^3^) are primarily used as wall materials, while a graphite coating (density 0.82 g/cm^3^) is exclusively used for PTW 31013. Similar to $P_{cel}$, $P_{wall}$ shows greater deviations from unity with decreasing sensitive volume. Notably, PTW 31013 displays a distinct trend, yielding a lower $P_{wall}$ than other chambers, likely due to the exclusive use of low-density graphite coating.

**Table 6:** Materials and density of the central electrodes and $P_{cel}$ values of the cylindrical ionization chambers as used in the different studies. The maximum difference was 1.0.

Chamber	Author	central electrode materials	$P_{cel}$	Maximum difference
PTW 30013	This study Baumann et al. Kretchmer et al.	Aluminum (2.70 g cm^−3^)	0.994–0.996	0.60%
PTW 31013	This study	Aluminum (2.70 g cm^−3^)	0.996–0.998	0.40%
PTW 31014	Kretchmer et al.	Aluminum (2.70 g cm^−3^)	0.994–0.998	0.60%
PTW 31021	Kretchmer et al.	Aluminum (2.70 g cm^−3^)	0.996–0.998	0.40%
PTW 31022	Kretchmer et al.	Aluminum (2.70 g cm^−3^)	0.999–1.006	0.60%
IBA FC65-G	Baumann et al.	Aluminum (2.70 g cm^−3^)	0.996–0.998	0.40%
NE 2571	Baumann et al. Kretchmer et al.	Aluminum (2.70 g cm^−3^)	0.997–0.998	0.30%
Exradin A1SL	Baumann et al.	C552 (1.76 g cm^−3^)	0.998–1.002	0.20%

**Table 7:** Materials and density of the chamber wall and $P_{wall}$ values of the cylindrical ionization chambers as used in the different studies. The maximum difference was 1.0.

Chamber	Author	wall materials	$P_{wall}$	Maximum difference
PTW 30013	This study Baumann et al. Kretchmer et al.	PMMA (1.19 g cm^−3^) Graphite (1.85 g cm^−3^)	0.986–0.993	1.40%
PTW 31013	This study	PMMA (1.19 g cm^−3^) Graphite Coating (0.82 g cm^−3^)	0.990–0.995	1.00%
PTW 31014	Kretchmer et al.	PMMA (1.19 g cm^−3^) Graphite (1.85 g cm^−3^)	0.986–0.989	1.40%
PTW 31021	Kretchmer et al.	PMMA (1.19 g cm^−3^) Graphite (1.85 g cm^−3^)	0.986–0.992	1.40%
PTW 31022	Kretchmer et al.	PMMA (1.19 g cm^−3^) Graphite (1.85 g cm^−3^)	0.981–0.991	1.90%
IBA FC65-G	Baumann et al.	PMMA (1.19 g cm^−3^) Graphite (1.85 g cm^−3^)	0.983–0.988	1.70%
NE 2571	Baumann et al. Kretchmer et al.	PMMA (1.19 g cm^−3^) Graphite (1.85 g cm^−3^)	0.983–0.988	1.70%
Exradin A1SL	Baumann et al.	C552 (1.76 g cm^−3^)	0.982–0.986	1.80%

### Monte Carlo $k_{Q}$ for monoenergetic proton beams of the Semiflex ionization chamber

**Fig. 6:**
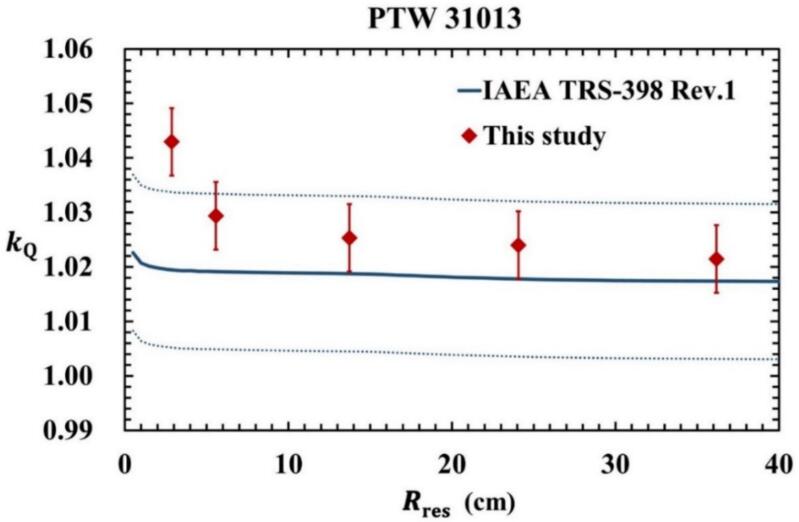
Comparing the $k_{Q}$ of PTW 31013 in TRS-398 Rev. 1 with the $k_{Q}$ determined from the $f_{Q}$ calculated in this study. The dotted line shows the uncertainty of $k_{Q}$ in TRS-398 Rev. 1.

Fig. 6 shows the $k_{Q}$ values provided in TRS-398 Rev. 1 and those calculated for PTW 31013 using the $f_{Q}$ calculated in this study. The error bars indicate standard uncertainties. The dotted line represents the uncertainty range of $k_{Q}$ in TRS-398 Rev. 1. For $R_{res}$ below 15 cm, the calculated $k_{Q}$ values deviated by up to 2.3% from the reference values currently in use. In contrast, for $R_{res}$ above 15 cm, the deviations were limited to 0.6% and remained within the stated uncertainty margins. Based on these findings, the use of cylindrical ionization chambers is recommended for $R_{res}$ above 15 cm, in agreement with TRS-398 Rev. 1 guidelines. A previous study reported a maximum deviation of 0.7% for PMMA wall materials compared to TRS-398 Rev. 1 data, with similar discrepancies observed by Palmans et al. [29]. Before the update, TRS-398 Rev. 1 included data for both PTW 31003 and an earlier version of PTW 31013, with a maximum reported difference of 0.5%. To the best of our knowledge, this is the first study to provide a calculated $k_{Q}$ for PTW 31013, highlighting the need for further detailed investigations.

Although this study was limited to monoenergetic proton beams and did not include evaluations under Spread-Out Bragg Peak (SOBP) or other clinical conditions, the findings remain highly relevant. TRS-398 Rev. 1 recommends absolute dose measurements under monoenergetic beam conditions as the standard for scanning proton beam dosimetry, which supports the validity of the chosen approach. While clinical practice often requires measurements under various SOBP and non-reference conditions, existing data on $f_{Q}$ values for such scenarios are limited. Only two previous studies, conducted by Gomà and Sterpin. [4] and Palmans et al. [30], reported evaluations at a single point within an SOBP and did not include detailed uncertainty analyses. Future studies should evaluate factors under SOBP conditions to bridge this gap and enhance the clinical applicability of the results.

The methodology and results presented in this study provide essential reference data that enhance the understanding of ionization chamber response in high-precision proton therapy. Furthermore, these findings are applicable to broader clinical contexts and contribute to reducing uncertainty while improving the safety and accuracy of proton dosimetry.

## Conclusion

To the best of our knowledge, this study is the first to calculate $f_{Q}$ and $P_{Q}$ for proton beams in various ionization chambers using MC code PHITS. The results extend existing MC simulation data for $k_{Q}$ in proton beams and provide essential data for uncertainty evaluation. These findings offer valuable reference data that may support further refinement of $k_{Q}$ and improve the precision of proton beam dosimetry.

## Declaration of competing interest

The authors declare that they have no known competing financial interests or personal relationships that could have appeared to influence the work reported in this paper.

## References

[1] International atomic energy agency; (2024). International Atomic Energy Agency. Absorbed Dose Determination in External Beam Radiotherapy. Rev..

[2] Salvat F. (2013). A generic algorithm for Monte Carlo simulation of proton transport. Nuclear Instruments and Methods in Physics Research Section B: Beam Interactions with Materials and Atoms.

[3] Agostinelli S., Allison J., Amako K., Apostolakis J., Araujo H., Arce P. (2003). Geant4—a simulation toolkit. Nuclear Instruments and Methods in Physics Research Section A: Accelerators, Spectrometers, Detectors and Associated Equipment.

[4] Gomà C., Sterpin E. (2019). Monte Carlo calculation of beam quality correction factors in proton beams using PENH. Physics in Medicine & Biology.

[5] Baumann K., Horst F., Zink K., Gomà C. (2019). Comparison of PENH, FLUKA, and Geant4/TOPAS for absorbed dose calculations in air cavities representing ionization chambers in high‐energy photon and proton beams. Med Phys.

[6] Baumann K.-S., Derksen L., Witt M., Michael Burg J., Engenhart-Cabillic R., Zink K. (2021). Monte Carlo calculation of beam quality correction factors in proton beams using FLUKA. Phys Med Biol.

[7] Baumann K.-S., Kaupa S., Bach C., Engenhart-Cabillic R., Zink K. (2020). Monte Carlo calculation of beam quality correction factors in proton beams using TOPAS/GEANT4. Phys Med Biol.

[8] Kretschmer J., Dulkys A., Brodbek L., Stelljes T.S., Looe H.K., Poppe B. (2020). Monte Carlo simulated beam quality and perturbation correction factors for ionization chambers in monoenergetic proton beams. Med Phys.

[9] Nagake Y., Yasui K., Ooe H., Ichihara M., Iwase K., Toshito T. (2024). Investigation of ionization chamber perturbation factors using proton beam and Fano cavity test for the Monte Carlo simulation code PHITS. Radiol Phys Technol.

[10] Baumann K.-S., Kaupa S., Bach C., Engenhart-Cabillic R., Zink K. (2021). Monte Carlo calculation of perturbation correction factors for air-filled ionization chambers in clinical proton beams using TOPAS/GEANT. Zeitschrift Für Medizinische Physik.

[11] Baumann K.-S., Gomà C., Wulff J., Kretschmer J., Zink K. (2023). Monte Carlo calculated ionization chamber correction factors in clinical proton beams - deriving uncertainties from published data. Physica Medica.

[12] Ferrari A., Sala P.R., Fasso A., Ranft J., Fluka: a, (2005). Multi-Particle Transport Code..

[13] Battistoni G., Bauer J., Boehlen T.T., Cerutti F., Chin M.P.W., Dos Santos A.R. (2016). The FLUKA Code: An Accurate Simulation Tool for Particle Therapy. Front. Oncol.

[14] Sato T., Iwamoto Y., Hashimoto S., Ogawa T., Furuta T., Abe S. (2018). Features of Particle and Heavy Ion Transport code System (PHITS) version 3.02. Journal of Nuclear Science and Technology.

[15] Sechopoulos I., Rogers D.W.O., Bazalova-Carter M., Bolch W.E., Heath E.C., McNitt-Gray M.F. (2018). RECORDS: improved Reporting of montE CarlO RaDiation transport Studies: Report of the AAPM Research Committee Task Group 268. Medical Physics.

[16] Sempau J., Andreo P., Aldaba J., Mazurier J., Salvat F. (2004). Electron beam quality correction factors for plane-parallel ionization chambers: Monte Carlo calculations using the PENELOPE system. Physics in Medicine & Biology.

[17] Andreo P., Burns D.T., Kapsch R.P., McEwen M., Vatnitsky S., Andersen C.E. (2020). Determination of consensus kQ values for megavoltage photon beams for the update of IAEA TRS-398. Phys Med Biol.

[18] Wulff J., Heverhagen J.T., Zink K. (2008). Monte-Carlo-based perturbation and beam quality correction factors for thimble ionization chambers in high-energy photon beams. Physics in Medicine & Biology.

[19] Zink K., Wulff J. (2008). Monte Carlo calculations of beam quality correction factors kQ for electron dosimetry with a parallel-plate Roos chamber. Physics in Medicine & Biology.

[20] Seltzer SM, Fernandez-Varea JM, Andreo P, Bergstrom PM, Burns DT, Krajcar Bronić I, et al. Key data for ionizing-radiation dosimetry: measurement standards and applications, ICRU Report 90.

[21] Takada K., Sato T., Kumada H., Koketsu J., Takei H., Sakurai H. (2017). Validation of the physical and RBE-weighted dose estimator based on PHITS coupled with a microdosimetric kinetic model for proton therapy. Journal of Radiation Research.

[22] Sato T., Furuta T., Liu Y., Naka S., Nagamori S., Kanai Y. (2021). Individual dosimetry system for targeted alpha therapy based on PHITS coupled with microdosimetric kinetic model. EJNMMI Physics.

[23] Iwamoto Y., Hashimoto S., Sato T., Matsuda N., Kunieda S., Çelik Y. (2022). Benchmark study of particle and heavy-ion transport code system using shielding integral benchmark archive and database for accelerator-shielding experiments. Journal of Nuclear Science and Technology.

[24] Shouop C.J.G., Mbembe S.M., Kamkumo C.T., Ateba J.F.B., Moyo M.N., Mekongtso E.J.N. (2022). Monte Carlo optimum management of 241Am/Be disused sealed radioactive sources. Scientific Reports.

[25] Boukhellout A., Ounoughi N., Kharfi F. (2022). MONTE-CARLO SIMULATION USING PHITS OF SECONDARY NEUTRONS PRODUCED IN-PATIENT DURING 16O ION THERAPY. Radiat Prot Dosimetry.

[26] Boudard A., Cugnon J., David J.-C., Leray S., Mancusi D. (2013). New potentialities of the Li\‘ege intranuclear cascade model for reactions induced by nucleons and light charged particles. Phys Rev C.

[27] Lynch G.R., Dahl O.I. (1991). Approximations to multiple Coulomb scattering. Nucl Instrum Meth B.

[28] Baumann K.-S., Derksen L., Witt M., Adeberg S., Zink K. (2023). The influence of different versions of FLUKA and GEANT4 on the calculation of response functions of ionization chambers in clinical proton beams. Phys Med Biol.

[29] Palmans H., Lourenço A., Medin J., Vatnitsky S., Andreo P. (2022). Current best estimates of beam quality correction factors for reference dosimetry of clinical proton beams. Phys Med Biol.

[30] Palmans H., Thomas R., Simon M., Duane S., Kacperek A., DuSautoy A. (2004). A small-body portable graphite calorimeter for dosimetry in low-energy clinical proton beams. Phys Med Biol.

